# COVID19, the pandemic which may exemplify a need for harm-reduction approaches to eating disorders: a reflection from a person living with an eating disorder

**DOI:** 10.1186/s40337-020-00306-3

**Published:** 2020-05-31

**Authors:** Margaret Janse van Rensburg

**Affiliations:** Carleton University, Ottawa, Canada

**Keywords:** Reflection, Harm reduction, Strengths-based approach

## Abstract

This reflective piece, written by a woman with an eating disorder aims to identify the impact of COVID-19 on persons living with eating disorders and provide a social justice approach as a resolution. The author identifies that eating disorder behaviors may be the only coping tool available for many persons with eating disorders during this time of uncertainty. While she acknowledges the risks associated with eating disorder behaviors, she identifies that this time of uncertainty may be a time to embrace harm-reduction in approaching the health and wellness of persons with eating disorders.

## Main text

Touyz, Lacey, & Hay [8] published the editorial “Eating disorders in the time of COVID-19”, which outlines the unique impact of COVID-19 for persons with eating disorders (EDs). However, they provide little suggestions for readers as to how this impact can be managed. This letter seeks to promote a harm-reductionist approach to EDs during this uncertain time.

I identify as a person who lives with an eating disorder (ED). First diagnosed at fifteen, I spent my adolescence and young adulthood in therapeutic settings seeking normalcy. I completed a plethora of programs, therapies, and experimental treatments for my ED. My symptoms have decreased markedly, but I still question ‘recovery’. I have just completed my Master of Social Work which has an ethical commitment to social justice. I believe a social justice approach to EDs would prioritize harm-reduction.

Touyz, Lacey, & Hay [8]‘s report of the impacts of COVID-19 on persons with EDs are accurate. For me, grocery shelves becoming empty of my staples caused me great anxiety. “How am I going to adapt my eating schedule?” I asked myself. Social isolation has meant that there was little available for me in terms of ‘crowding out’ the ED with other meaningful activities. While I claim no expertise in this area, I can also imagine that there are a plethora of reasons why persons with EDs may be at higher risk of contracting COVID-19, including persons with EDs having more hand-to-mouth behaviors, having an inability to isolate, and having more contact with health care settings that have COVID-19 outbreaks. I can also extrapolate that COVID-19 has been a real nightmare for care providers, who may be unable to provide, or be forced to adapt, recovery-centered support.

Furthermore, COVID-19 may leave persons with EDs in especially vulnerable places. Persons with EDs are often stigmatized and seen by society as responsible for their eating disorders [5]. In this pandemic, all people are coping the best way they know how. Persons with EDs have unique, and sometimes dangerous coping mechanisms. The elevated mortality associated with EDs, especially those from suiciding are particularly concerning [1, 2, 9].

Can we use COVID-19 as an opportunity to promote social justice in the field of ED research and practice? Current research surrounding EDs as social justice issues focus on prevention [6], calling for a resolution in “target [ing] the systems that are contributing to the problem and not the individual who is affected” ([7], p. 140). In my opinion, during COVID-19, the task of recovery may be unattainable or unpractical given contextual circumstances. Furthermore, targeting systems may leave persons with EDs in precarious circumstances for their health and wellbeing. Logan & Marlatt [4] identify that harm reduction approaches seek to reduce the negative impacts of dangerous behaviors. A social justice approach therefore must prioritize harm-reduction of ED behaviors: the negative physiological, psychological, and social impacts of behaviors.

Studies are increasingly identifying that there are external factors which remove personal responsibility from ED development and maintenance, including genetic and environmental risk factors [3, 10]. As Touyz, Lacey, & Hay [8] acknowledged, the environmental circumstances of COVID-19 may increase ED behaviors. During this time, persons with EDs may face ostracization by friends and families, who may oppose them using an ED as a coping tool. These skills have potential to harm, but may also act as a lifesaver to the person with an ED, giving them purpose and the ability to navigate this exceptional time. This is a time where a person’s livelihood may depend on maintaining, rather than ceasing, certain ED behaviors.

During this pandemic, I continue to allow myself to count, calculate, and control my eating and exercise. I binge nightly without guilt. I ensure my weight remains healthy and stable, I do not vomit anymore, and I take my medications daily. Maintaining my ED, in as healthful way as possible, is a coping strategy which gives me control during a time when I have lost complete control.

I am careful not to prescribe my own practice. A harm-reductionist approach must be individual, especially given the differing circumstances of each person, taking into each person’s intersectional circumstances. Table 1 describes the unique differences between a traditional treatment approach and a harm-reductionist approach to eating disorders.

**Table 1:** The difference between a harm-reductionist approach and a traditional treatment approach

	Traditional Treatment Approach	Harm-Reductionist Approach
Goal of Treatment	Reduction, cessation, and/or abstinence of ED behavior.	Decrease likelihood of mortality. Behaviors can be maintained. The goal is to increase the safety of ED behavior.
Goal of Treatment outcome	Find a *life worth living* outside an ED. Restore a ‘normal’ eating pattern, where a normative pattern upholds colonial and capitalist structures of oppression.	Find *a life worth living* while living with an ED. Identify that normative eating is not necessary or possible, and that the person can find their own normal, and that their normal can shift over time.
Perspective of ED	Pathological: ED is seen as extrinsic to the person, and is often compared to cancer or an abusive partner. Treatment team identifies that behaviors are harmful and must be ceased to increase health and well-being.	Strengths-based: ED is viewed as a coping mechanism which naturally developed given their biological susceptibility and the environment they live in. Allows persons with ED to set boundaries about what behaviors they are not willing to give up at that time. The person has access to education on the harms of behaviors and on how to reduce the harms of these behaviors if they are maintained.
Power	Top-down approach to treatment where the clinician is the expert of the clients health. Nutrition and weight goals are prescribed to person living with an ED, tracked by the clinician who has access to restricted client information. Person living with an ED may limit the information they share in fear of judgment and suggestions of change.	Person-centered and strengths-based approach where the person with the ED is the true expert of their health. Goals and information are decided and tracked by the person living with an ED. They can share this information with a clinician or similar, if they have access, when they trust that there will be no judgment or directions to change the behavior.
Family approaches	Family is recruited as an extension of a treatment team. They may monitor behavior, make rules around food and exercise, and communicate with a treatment team about *progress* or symptoms of the person with an ED.	Any communication with family by a treatment team is focused on conflict prevention and resolution. Family is taught to set boundaries, about non-judgmental approaches, and encouraged to focus on their own well-being.
Research	Group-based research is seen as the gold standard for identifying evidence-based approaches.	Evidence is based on the individual. A person is their own case study, setting their own baseline, finding techniques that maintain their health and well-being based on success and usefulness in the past.

It is therefore evident that during this time, persons with EDs, families, friends, clinicians, and researchers must identify if harm-reduction can be an alternative, and potentially temporary, focus. This approach seeks to increase self-determination and promote social justice on an individual level. Rather than giving up completely, or forcing ED recovery, a harm-reductionist approach embraces the uncertainty of our times, and promotes a strengths-based dialectic perspective of EDs.

## Data Availability

N/A

## Abbreviations

ED: Eating Disorder
EDs: Eating Disorders
